# High-field MRI findings in epileptic dogs with a normal inter-ictal neurological examination

**DOI:** 10.3389/fvets.2024.1507861

**Published:** 2025-01-15

**Authors:** Stephanie Phillipps, Rita Goncalves

**Affiliations:** Small Animal Teaching Hospital, Institute of Infection, Veterinary and Ecological Sciences, University of Liverpool, Neston, United Kingdom

**Keywords:** dog, epilepsy, structural, normal, MRI, inter-ictal

## Abstract

**Introduction:**

Epilepsy is one of the most common chronic neurological conditions affecting dogs. Previous research exploring the likelihood of a structural cause of epilepsy specifically in dogs with a normal inter-ictal examination is limited to a small population of dogs using low-field MRI. The aims of this study were to establish high-field (1.0T and 1.5T) MRI findings in dogs presenting with epileptic seizures and a normal inter-ictal examination.

**Methods:**

Medical records were retrospectively searched for dogs presenting with at least two epileptic seizure events more than 24 h apart. To be included in the study, patients had to have a normal neurological examination, high-field MRI of the brain and have had metabolic and toxic causes excluded.

**Results:**

Four hundred and twelve dogs were eligible for inclusion. Crossbreeds were most commonly affected (*n* = 63, 15.3%) followed by Border collies (*n* = 39, 9.5%) and Labrador retrievers (*n* = 26, 6.3%). Seventy-six dogs (18.5%) had abnormalities detected on MRI, 60 (78.9%) of which were considered to be incidental. Overall, 16 dogs (3.9%) had a structural cause of their epileptic seizures including neoplasia (*n* = 13, 81.3%), anomalous (*n* = 2, 12.5%) and meningoencephalitis of unknown origin (MUO) (*n* = 1, 6.3%). When split into age group at first epileptic seizure structural lesions were documented in 0/66 dogs aged <1 year, 4/256 (1.6%) dogs aged ≥1 year ≤6 years (three neoplastic and one anomalous), 3/51 (5.9%) aged >6 years ≤8 years (two neoplastic and one MUO), and 9/39 (23.1%) dogs aged >8 years (eight neoplastic, one anomalous). Multivariate analysis identified two risk factors for structural disease: increasing age at first epileptic seizure (*p* < 0.001, OR = 4.390, CI 2.338–8.072) and a history of status epilepticus (*p* = 0.049, OR = 4.389, CI 1.010–19.078).

**Discussion:**

Structural lesions are an uncommon cause of epilepsy at any age in dogs with a normal inter-ictal examination.

## Introduction

The worldwide lifetime prevalence of active epilepsy (defined as ongoing seizures or seizures requiring ongoing treatment) in humans is estimated to be 0.76%, with around 50 million people worldwide affected (1). In dogs, the incidence of at least one epileptic seizure event is estimated to be 0.82% (2), with epilepsy of unknown origin diagnosed in approximately 0.62% of dogs attending UK primary care practices (3). With an estimated 11 million dogs owned in the UK alone (4), this equates to over 90,000 dogs experiencing a single epileptic seizure in a one-year period and 68,000 diagnosed with epilepsy of unknown origin. Unsurprisingly then, epileptic seizure disorders are the most common presenting neurological complaint in dogs (5), and will be encountered regularly by most small animal clinicians.

Epileptic seizures may be secondary to a number of different disease processes, including metabolic or toxic aetiologies (termed ‘reactive seizures’), structural, and idiopathic (genetic, suspected genetic and unknown) causes (6). Current recommendations made by the International Veterinary Epilepsy Task Force (IVETF) are that dogs aged below 6 months or above 6 years of age at the onset of their first epileptic seizure should have magnetic resonance imaging (MRI) of the brain performed (6). The presence of an abnormal inter-ictal neurological examination has been demonstrated to be strongly associated with an increased risk of structural disease as the cause of epileptic seizures in dogs (7), however IVETF imaging recommendations relating to age are regardless of the outcome of the inter-ictal neurological examination. These guidelines were based on previous studies which suggested statistically significant increase in risk of a structural lesion outside of these age limits (8). As the growing body of research into canine epilepsy continues to expand however, the scientific rigor on which these recommendations were made has been called into question. Some of the studies cited in reaching this age cut off did not differentiate between dogs with normal or abnormal inter-ictal neurological examinations (8, 9) or did not require reliable neurological examinations to have been performed in all patients at all (10). Other studies also included neurologically abnormal dogs in the ‘neurologically normal’ population, such as mentation changes and cervical hyperaesthesia (11) or post-ictal changes and abnormalities likely attributed to anti-seizure drugs (ASDs) (12) included as neurologically normal. Although the overall reported incidence of structural disease in some of the papers used in the recommendations is high (10, 13), if dogs with abnormal neurological examinations are excluded then structural disease varies from 5.3% (14) to 31% (13) rather than the 26 and 59% reported in the IVETF guidelines (6). Additionally, many of these studies used small patient populations, which are even smaller when only neurologically normal animals are included.

More recently, a large study of 900 dogs undergoing MRI at multiple institutions to investigate the cause of recurrent seizure disorders found statistically significant association between the diagnosis of idiopathic epilepsy and the patient age group 6 months to 6 years (15). Again, patients did not have to be neurologically normal in between epileptic seizures in order to be included in the study and 70.9% of dogs aged over 6 years had structural lesions found on MRI, but only 3.4% of dogs less than 6 months of age. With the increasing availability of high-field MRI, it has been postulated that more subtle lesions may be found due to the improved spatial and image resolution compared to low-field scans (16). Additionally, earlier lesions may be discovered, particularly given how the IVETF recommendations may affect which animals are recommended to have an MRI scan performed.

Recommendations by the IVETF are also to perform MRI scans in all patients that experience cluster seizures or status epilepticus (6). Previously reported prevalence of cluster seizures in a population of 407 dogs diagnosed with idiopathic epilepsy presenting to a referral institution is 41% and status epilepticus in 10% (17). In a recent study investigating risk factors relating to outcome of patients with cluster seizures or status epilepticus, 41/93 (44.1%) were diagnosed with idiopathic epilepsy (18) and 50/124 (40.3%) in another focussing on status epilepticus alone (19). These findings are fairly consistent with previous literature documenting idiopathic epilepsy as the cause of 40.3% of cluster seizures and between 13.2–37.5% of status epilepticus (7, 20). None of these studies assessed risk in only patients with a normal neurological examination that were yet to be diagnosed with the cause of their epileptic seizures, and so it remains unknown whether the presence of cluster seizures or status epilepticus increases the risk of structural disease in this patient population.

Performing a brain MRI results in significant financial cost for pet owners and also requires general anaesthesia or profound sedation, which are not risk-free procedures (21). Additionally, there may be limited access to this imaging modality depending on geographic location as reported in human medicine (22). With the cost of living crisis and the increasing demand from pet owners that veterinarians offer rational diagnostic tests which are specific to that patient, rather than recommending so called ‘gold standard’ workup in every case, there is a need for up-to-date, accurate information on the diagnostic utility of brain MRI in epileptic patients with a normal neurological examination.

The aims of this study were therefore to (1) report the high-field MRI findings in a large group of epileptic dogs with a normal inter-ictal neurological examination, (2) establish the prevalence of structural disease within this patient population and any association with signalment or seizure type.

## Materials and methods

Study design was retrospective. Digital medical records from the University of Liverpool Small Animal Teaching Hospital were searched for dogs presenting for investigation of epileptic seizures between December 2008 and May 2024. Inclusion criteria required that medical records were complete for review (clinical history, treatment while hospitalized, signalment), that dogs had experienced more than one seizure event (>24 h apart), had undergone a complete neurological examination by a diplomate of the European College of Veterinary Neurology (ECVN) and/or ECVN resident, as well as MRI of the brain and having had metabolic and toxic causes of seizures excluded through clinical history taking and routine bloodwork (haematology, biochemistry, bile acid stimulation test as had been deemed necessary by the clinician responsible for the case). Dogs were excluded if a full neurological examination could not be performed, if there were any abnormalities found on neurological examination (including bilaterally symmetrical signs that may have been attributable to post-ictal changes or side effects of commonly prescribed ASDs), if it was unclear from the history whether epileptic seizures or other paroxysmal events were responsible for the clinical signs, if the clinical history provided by the owner was consistent with neurological abnormalities other than epileptic seizure activity and if medical data or MRI were not available for review. The study was approved by the University of Liverpool Veterinary Research Ethics Committee (reference number VREC752).

Information on signalment, clinical history, neurological examination findings, clinicopathological findings, MRI findings, treatment administered, outcome and follow-up information were collected from the records. Follow-up information was recorded at re-examinations. Breeds were recorded both individually and purebreds were also grouped by skull conformation type. For statistical analysis, breeds with more than 10 animals represented were analysed separately and all other dogs (including crossbreeds) were grouped as ‘other’. Dogs were grouped by age at the time of their MRI as follows: <6 months, ≥6 months <1 year, ≥1 year ≤6 years, >6 years ≤8 years and > 8 years. The same age grouping was also performed for age at onset of seizures. Based on the owner’s, referring veterinarian’s description or hospital records, seizure types were grouped into generalized epileptic seizures only, focal epileptic seizures only, or both generalised and focal epileptic seizures. Any cases which experienced focal epileptic seizures with secondary genersalisation were included in the latter group. Video records were utilised where available by the attending veterinarian to characterise epileptic seizure type. Presence of cluster seizures (>1 seizure in a 24 h period) and status epilepticus (a single event lasting >5 min or two events without regaining full consciousness in between) were also recorded. Cerebrospinal fluid (CSF) analysis was evaluated where available but was not a prerequisite for inclusion in the study.

Magnetic resonance images were acquired from December 2008 to July 2015 with a 1.0T MRI scanner (Magnetom 1.0T; Siemens, Forchheim, Germany) and from August 2015 to May 2024 with a 1.5T scanner (Ingenia 1.5TCX; Philips Medical Systems, Eindhoven, the Netherlands). As the 1.0T scanner is a super conducting magnet all images were considered high field (23). Sequences performed included a minimum of T2-weighted images (T2W) (in transverse and sagittal planes at least), fluid attenuation inversion recovery (FLAIR) and pre- and post-contrast (intravenous injection of 0.1 mmol/kg of gadopentetate dimeglubine) T1-weighted images (T1W) in the transverse plane. Images were reviewed by a board-certified diagnostic imager and a board certified and/or a residency trained neurologist. A diagnosis of peri-ictal change was made based on the presence of bilaterally symmetrical T2W/FLAIR hyperintensity of the hippocampus, piriform lobes +/− cingulate gyri (24). Cerebrospinal fluid collection was performed under the same general anaesthesia and analysis comprised a red blood cell (RBC) count and total nucleated cell concentration (TNCC), protein concentration measurement and a cytological examination with a differential cell count. Normal CSF TNCC was defined as less than 5 cells/μl and normal CSF total protein as less than 0.45 g/L (25).

Descriptive statistics were performed using Excel (Microsoft Corportation, Redmond, Washington, USA 2020).

Statistical analysis was performed using the software SPSS 27.0 (SPSS Inc., Chicago, Illinois, USA). Continuous data were assessed for normality using the Shapiro–Wilk test. For normally distributed data, the range and mean values are presented and in abnormally distributed sets, the range, median value and interquartile range (IQR) are provided. Excel data was then imported into SPSS for statistical analysis.

Binary logistic regression modelling was utilised to evaluate the univariable associations between risk factors (age group at first epileptic seizure, length of time between epileptic seizure onset and MRI, breed, sex, neuter status, generalised vs. focal seizures, presence of cluster seizures and status epilepticus) and structural abnormalities on MRI. Before multivariable analysis, all variables were assessed for correlation using Spearman’s rank correlation coefficients. If Spearman’s rank correlation coefficient was >0.8, the most statistically significant or biologically plausible variable was selected. Independent variables demonstrating liberal association (*p* < 0.2) were taken forward for multivariate modelling in which statistical significance was set at *p* < 0.05.

## Results

Four hundred and sixteen dogs met the inclusion criteria. Four dogs were excluded from analysis because the results of their MRI could not definitively be categorised as either causative of epileptic seizures or an incidental finding (two dogs with chronic lacunar infarcts, one dog with a small meningeal nodule which was stable at follow-up MRI 7 months later and one dog with leukoaraiosis). Four hundred and twelve dogs were therefore included in the study. Crossbreeds were most commonly affected (63/412, 15.3%), and the Border collie (39/412, 9.5%), Labrador retriever (26/4132, 6.3%) and Cavalier King Charles spaniels (19/412, 4.6%) were the most commonly reported purebreds (Table 1). Ninety-eight dogs (23.8.0%) were brachycephalic, 214 (51.9%) were mesocephalic and 39 (9.5%) were dolichocephalic. One hundred and thirty-one (31.8%) dogs were female (85/131, 64.9% neutered) and 281 (68.2%) were male (175/281, 62.3% neutered).

**Table 1:** Most frequent breeds of dog with epilepsy and a normal inter-ictal neurological examination.

Breed	Number (%)	No (%) with structural disease
Crossbreed	63 (15.3)	2 (3.2)
Border collie	39 (9.5)	0 (0)
Labrador retriever	26 (6.3)	0 (0)
Cavalier King Charles spaniel	19 (4.6)	0 (0)
Staffordshire bull terrier	18 (4.4)	4 (22.2)
Cocker spaniel	14 (3.4)	0 (0)
French bulldog	14 (3.4)	1 (7.1)
Pug	13 (3.2)	0 (0)
Border terrier	12 (2.9)	4 (33.3)
English springer spaniel	12 (2.9)	0 (0)
Chihuahua	11 (2.7)	0 (0)
Other purebreed	171 (41.5)	6 (3.5)

Median age at diagnosis was 51 months (IQR 25–76 months, range 2–187). Age group split at diagnosis was <6 months 15 dogs (3.6%), ≥6 months <1y 26 dogs (6.3%), ≥1 year ≤6 years 254 dogs (61.7%), >6 years ≤8 years 66 dogs (16.0%), > 8 years 51 dogs (12.4%).

The median age at first epileptic seizure was 40 months (IQR 19–70, range 1–187). Age group split at first epileptic seizure was <6 months 25 dogs (6.1%), ≥6 months <1y 41 dogs (10.0%), ≥1 year ≤6 years 256 dogs (62.1%), >6 years ≤8 years 51 dogs (12.4%), >8 years 39 dogs (9.5%).

Two hundred and thirty-six (57.3%) of dogs had MRI performed with the 1.5T magnet and 176 (42.7%) with the 1T magnet. Seventy-six (18.4%) of the 412 dogs had abnormalities detected on MRI, 60 (78.9%) of which were considered to be incidental. Incidental findings included Chiari-like malformation (*n* = 30, 50%), otitis (*n* = 20, 33.3%) (15 otitis media, five otitis externa), syringohydromyelia (*n* = 13, 21.7%) dilation of the ventricular system (*n* = 6, 10%), peri-ictal change (*n* = 5, 8.3%), mild cortical atrophy (*n* = 4, 6.7%), atlantoaxial band (*n* = 3, 5%), absent septum pellucidum (*n* = 3, 5%) mild supracollicular fluid accumulation (*n* = 2, 3.3%) and one each (1.7%) of: caudal calvarial malformation, ear mass (not affecting the para aural tissues), vascular malformation in the fourth ventricle (extra-parenchymal and not resulting in hydrocephalus), dental disease, atlantoaxial band, pterygoid muscle lesion and temporal muscle lesion.

Sixteen (3.8%) dogs had clinically significant structural changes on MRI: 13 (81.3%) were suspected neoplastic (nine suspected gliomas, two meningiomas, one suspected histiocytic sarcoma and one calvarial mass), two (12.5%) anomalous conditions (one cortical dysplasia and the other porencephaly) and one (6.3%) inflammatory disease (meningoencephalitis of unknown origin). Breeds affected by structural lesions included four Staffordshire bull terriers (all gliomas), four Border terriers (all gliomas), two crossbreeds (one suspected histiocytic sarcoma, one porencephaly), one Old English sheepdog (cortical dysplasia), one French bulldog (MUO), one Soft coated Wheaten terrier (suspected meningioma), one Boxer (glioma), one Miniature Poodle (meningioma) and one English bulldog (glioma) (Table 1).

When split into age at diagnosis groups, the distribution of structural causes of epileptic seizures was as follows: 0/15 (0%) dogs <6 months, 0/26 (0%) dogs ≥6 months <1y, 4/254 (1.6%) dogs ≥1 year ≤6 years (three neoplastic and one anomalous), 3/66 dogs (4.5%) >6 years ≤8 years (two neoplastic and one inflammatory), and 19/51 (17.6%) dogs >8 years (eight neoplastic and one anomalous).

When split into age at first epileptic seizure groups, the distribution of structural causes of epileptic seizures was as follows: 0/25 (0%) dogs <6 months, 0/41 (0%) dogs ≥6 months <1y, 4/256 (1.6%) dogs ≥1 year ≤6 years (three neoplastic and one anomalous), 3/51 (5.9%) dogs >6 years ≤8 years (one inflammatory and two neoplastic), 9/39 (23.1%) dogs >8 years (eight neoplastic and one anomalous) (Table 2).

**Table 2:** Diagnosis of structural disease in dogs with epilepsy and a normal inter-ictal neurological examination.

Age at First Seizure	No. (%) with structural disease	Disease type
<6 months	0/25 (0)	
≥6 months <1 year	0/41 (0)	
≥1 year ≤6 years	4/256 (1.6%)	3 neoplasia, 1 anomalous
>6 years ≤8 years	3/51 (5.9%)	2 neoplasia, 1 inflammatory
>8 years	9/39 (23.1%)	8 neoplasia, 1 anomalous

Overall, the median time between the onset of epileptic seizures and the time of diagnosis was 2.5 months (range 0.1–86 months, IQR 1–7 months), but in the structural group it was 1 month (range 0.1–20 months, IQR 0.2–1.6 months). The majority of patients with a structural cause of epileptic seizures had been experiencing epileptic seizures for 2 months or less (13/16, 87.5%), however two patients diagnosed with intracranial neoplasia had experienced epileptic seizures for a longer duration—one for 6 months and one for 20 months. The patient with the history of epileptic seizures of over 1 year experienced a single epileptic seizure 20 months prior to diagnosis, but no further epileptic seizures until the 3 months prior to diagnosis.

Two hundred and eighty-three (68.7%) dogs had generalised epileptic seizures only, 68 (16.5%) had focal epileptic seizures only and 61 (14.8%) dogs had both generalised and focal epileptic seizures recorded. One hundred and seventy-four (42.1%) dogs had a history of cluster seizures, including 164/396 (41.4%) dogs with no structural cause of epileptic seizures and 10/16 (62.5%) dogs with structural disease. Thirty-three dogs had experienced status epilepticus including 30/396 (7.6%) dogs with no structural cause of their epileptic seizures and 4/16 (25%) dogs with structural disease.

Cerebrospinal fluid was sampled in 274 (66.5%) dogs and was abnormal in 20 (7.3%) of sampled dogs, though blood contamination significant to preclude meaningful analysis was present in seven (35%) of these cases, leaving 13 animals with abnormal CSF results. Blood contamination was attributed as the cause of abnormal CSF findings in 2/13 (15.4%) of these dogs (RBC 2920 with a TNCC of 8 cells/μl in one dog with a glioma and RBC 9280 with a TNCC of 7 in a dog diagnosed with idiopathic epilepsy). In the remaining 11 dogs, TNCC was increased in nine (81.8%) cases (median 8 cells/μl, range 6–18 cells/μl). The highest count of 18 cells/μl was found in a dog with peri-ictal changes on MRI. All 11 of these dogs with an elevated TNCC were ultimately diagnosed with idiopathic epilepsy, received no immunosuppressive medications and had no neurological abnormalities at follow up. Abnormal protein concentration was reported in four (30.8%) of the dogs with abnormal CSF results (median 0.5 g/L, range 0.46–0.58 g/L). The dog with the highest total protein result of 0.58 g/L was diagnosed with idiopathic epilepsy and concurrent Chiari-like malformation and syringohydromyelia. One dogs with elevated total protein was diagnosed with a structural cause of their epilepsy (porencephaly).

The Spearman’s rank correlation coefficient between age at diagnosis and age at first epileptic seizure was 0.847 so only age at first epileptic seizure was included in the logistic regression model. On univariable analysis, breed, age at first epileptic seizure, the presence of cluster seizures and the presence of status epilepticus were associated with a diagnosis of structural disease. Only two variables remained significant at the multivariate level: age at first epileptic seizure was associated with a diagnosis of structural disease, with risk increasing as age group increased (*p* < 0.001, OR = 4.390, CI 2.388–8.072); history of status epilepticus was also associated with an increased risk of a diagnosis of structural disease (*p* = 0.049, OR = 4.389, CI 1.010–19.078).

## Discussion

From a total of 412 dogs that underwent an MRI of the brain to investigate causes of epilepsy with a normal inter-ictal neurological examination, 16 (3.9%) had a structural cause of their epileptic seizures. This is considerably less than that previously reported in the literature of 11.8–31% (11–13). Patients with structural disease tended to be older, with no animals under the age of one having a structural cause of their epileptic seizures, consistent with those findings of Bush et al. (11). There were only 66 patients less than 1 year old at the time of the onset of their epileptic seizures in the current study, which may explain the low numbers, however in a previous study looking specifically at the cause of epileptic seizures in 136 dogs under 1 year of age, of those with a normal neurological examination, only 6/114 (5.3%) had a structural cause of their epileptic seizures (14). Juvenile epilepsy has previously been thought to be more likely secondary to structural disease (6) including congenital brain malformations (14). Our findings in conjunction with previously published works however indicate that young patients with a normal inter-ictal neurological examination are most commonly diagnosed with idiopathic epilepsy, with other causes very uncommon. MRI is recommended early in the course of epilepsy in young children, as imaging changes secondary to ongoing epileptic seizure activity may complicate the diagnosis of lesions that may respond well to surgical management (22). In veterinary medicine, although surgical management of epilepsy has been described (26), this is currently rarely offered, and therefore performing MRI in these cases, unlike in human medicine, is mainly to exclude structural causes rather than for surgical assessment and planning and the human recommendations are therefore not applicable.

The proportion of patients with structural disease increased from 1 year of age, with patients over 8 years of age at the onset of their epileptic seizures having the highest proportion of structural disease (*n* = 16, 23.1%). This finding is consistent with previously published work indicating that older animals with a normal inter-ictal neurological examination are at increased risk of structural disease compared with younger animals (12, 13). Within our patient population however, the risk of structural disease in dogs over 6 years of age was 13.3% (12/90) which is lower than the previously reported 26.7% in dogs aged over six (12) and 31% in dogs aged seven or older (13). This likely reflects the more rigorous exclusion of dogs with inter-ictal abnormalities in the current study which is unique in the methodology compared to these previous works.

Full neurological examination cannot exclude significant structural disease, particularly in so-called ‘silent’ areas of the brain such as the olfactory region and piriform lobes (27). The most common structural lesion in this patient population was neoplasia, affecting 13/16 (81.3%) of patients with structural disease. Slowly progressive disease processes such as slow-growing neoplasms may be particularly challenging to detect clinically given the brain’s remarkable adaptation to slow change (28). These disease processes are more likely to affect the middle-aged to older canine population (29), which reflects the recommendations that animals over a certain age may benefit from advanced imaging to exclude structural lesions. It is important to appreciate however that 30/39 of dogs (76.9%) aged over 8 years old at the time of their first epileptic seizure in this study were diagnosed with epilepsy of unknown cause. In human medicine, the risk of developing epilepsy is bimodally distributed, with children aged <1 year of age having the highest risk, normalising to adult values by the age of 10 and then increasing again at around 60 years of age, with the highest risk >85 years (30). Our results suggest that geriatric onset epilepsy is also common in dogs, and that new onset epileptic seizures in an older pet should not be met with fear of a guarded prognosis in animals that are neurologically normal. Although it is sensible to discuss the merits of MRI given the relative risk of clinically silent structural lesions in older dogs, most of these patients will have a normal scan. Neurological disorders are among the top three causes of death in dogs in the UK (31), and this may be unwittingly biased by clinicians unduly considering neoplasia as the only likely cause of clinical signs, with its associated guarded prognosis. Our hope is that this study aids understanding of the importance of a full neurological examination and thorough history taking when considering most likely differential diagnoses in these patients, and that this will result in logical and rational decision making regarding further workup and treatment.

Of the 12 Border terriers included in our population, four (33.3%) had structural lesions and of the 18 Staffordshire bull terriers, four (22.2%) has structural lesions, with all lesions suspected to be neoplastic (gliomas). These breeds were also in the top three most common breeds with intra-axial neoplasia as a cause of their seizures in the study examining causes of epileptic seizures in 900 dogs, alongside boxers (15). A previous study recommended that all Boxer dogs that experience epileptic seizures should undergo an MRI of the brain due to a high prevalence of structural disease as the cause of epileptic seizures in this hospital population (32). There is incomplete information on the number of patients with a normal neurological examination in this study, but a high proportion (at least 44/74 dogs) had an abnormal examination. There were only six Boxers in our population, one (16.7%) with a structural lesion. Although care must be taken not to give undue significance to these results given the lack of significant association between breed and structural disease, the relatively higher percentage of dogs with structural abnormalities within these breeds may be worthy of further exploration with a larger study.

Occurrence of status epilepticus was associated with an increased risk of a structural lesions in our patient population. Although status epilepticus can be secondary to any cause of epileptic seizures (18–20, 33), a region of abnormal parenchyma may logically prevent intrinsic inhibitory neural networks from preventing further seizure propagation. Additionally, neoplasia may lead to upregulation of P-glycoprotein with resultant decreased concentrations of ASDs reaching their therapeutic targets in the brain, exacerbating status epilepticus (33). It is possible that diagnostic yield of performing brain MRI for the investigation of epileptic seizures in patients with a normal inter-ictal examination may therefore be greater in patients that are older at the onset of their epileptic seizures and have experienced status epilepticus.

Incidental findings on MRI were relatively common, with 60/412 patients (14.6%) having abnormalities not expected to cause epileptic seizures reported. MRI is a highly sensitive diagnostic imaging technique, capable of lesion detection with high sensitivity (34). Despite the use of high-field MRI in the current study, the proportion of dogs diagnosed with a structural lesion was less than that in the comparative low-field study. This likely reflects the rigorous exclusion of neurologically abnormal animals in the current study design, however the improved resolution of 1 and 1.5T images compared to the previous 0.3T study might also result in increased confidence in determining incidental from causative findings (35). It is possible however that smaller anatomical variations such as cortical dysplasia and early changes such as focal hippocampal sclerosis may be better visualised with 3T images or super high-field MRI (34), particularly given the small volume of the canine brain compared to humans.

Limitations of the current study include the retrospective nature which prevented individual case follow-up. Disease that was microscopic at the time of diagnosis could therefore not be completely excluded, for example paraneoplastic causes. Although MRI protocol was consistent throughout the study period at our institution, many patients did not undergo CSF collection and analysis. Cerebrospinal fluid sampling has been reported to be very safe in dogs (36), however the diagnostic utility in dogs with a normal inter-ictal examination is low (37, 38). Additionally, CSF abnormalities may be secondary to epileptic seizures themselves, rather than a marker of underlying aetiology (39). Although inflammatory disease cannot be completely excluded in the patients without CSF analysis, the authors propose that the diagnosis would be unlikely to be changed in these patients given the low value of this test in dogs with normal neurological examinations cited in other works. Finally, the diagnosis of epileptic seizure activity largely relied on owners descriptions of events. Electroencephalography (EEG) was not performed in any cases and although video footage was also reviewed where possible, cases of other types of paroxysmal event such as paroxysmal dyskinesia cannot be excluded (40). The study period spanned over nearly two decades and paroxysmal dyskinesia in particular is becoming more frequently recognised in veterinary neurology across many breeds of dog (41, 42). As paroxysmal dyskinesia episodes are rarely associated with structural disease, this may falsely lower the percentage of animals with no structural lesions on MRI if these cases are included. As our understanding of these conditions deepens, better testing to increase confidence in episode type (epileptic seizures vs. other) might help this diagnostic conundrum. Currently although frequently reported to be feasible in practice (43), few centres routinely offer EEG as part of the epileptic seizure workup.

## Conclusion

This study found that the most common cause of epileptic seizures in dogs with a normal inter-ictal neurological examination is epilepsy of unknown cause/idiopathic epilepsy, regardless of the age of onset of epileptic seizures. Although a normal neurological examination cannot completely exclude significant structural disease, most animals presenting with epileptic seizures and a normal inter-ictal examination will have a normal MRI scan. The age at diagnosis was associated with a significant increase in risk of a structural lesion in the over 8 years group, as did the presence of status epilepticus. It may be pertinent therefore to consider risk on a patient–patient basis when recommending an MRI scan in these cases.

## Data Availability

The raw data supporting the conclusions of this article will be made available by the authors, without undue reservation.

## References

[1] World Health Organisation (2019). Epilepsy: a public health imperative. Available at: https://www.who.int/publications/i/item/epilepsy-a-public-health-imperative (Accessed December 8, 2021).

[2] Erlen A Potschka H Volk HA Sauter-Louis C O'Neill DG. Seizure occurrence in dogs under primary veterinary care in the UK: prevalence and risk factors. J Vet Intern Med. (2018) 32:1665–76. doi: 10.1111/jvim.15290, PMID: 30216557 PMC6189390

[3] Kearsley-Fleet L O'Neill DG Volk HA Church DB Brodbelt DC. Prevalence and risk factors for canine epilepsy of unknown origin in the UK. Vet Rec. (2013) 172:338. doi: 10.1136/vr.101133, PMID: 23300065

[4] PDSA (2023). PDSA Animal Wellbeing (PAW) Report 2023. Available at: https://www.pdsa.org.uk/what-we-do/pdsa-animal-wellbeing-report/paw-report-2023 (Accessed September 1, 2023).

[5] Licht BG Lichgt MH Harper KM Lin S Curtin JJ Hyson LL. Clinical presentations of naturally occurring canine seizures: similarities to human seizures. Epilepsy & behavior. (2002). 3, 460–70. 12609269 10.1016/s1525-5050(02)00523-1

[6] De Risio L Bhatti S Muñana K Penderis J Stein V Tipold A. International veterinary epilepsy task force consensus proposal: diagnostic approach to epilepsy in dogs. BMC Vet Res. (2015) 11:148. doi: 10.1186/s12917-015-0462-1, PMID: 26316175 PMC4552251

[7] Armaşu M Packer RMA Cook S Solcan G Volk HA. An exploratory study using a statistical approach as a platform for clinical reasoning in canine epilepsy. Vet J. (2014) 202:292–6. doi: 10.1016/j.tvjl.2014.08.008, PMID: 25241948

[8] Podell M Fenner WR Powers JD. Seizure classification in dogs from a nonreferral-based population. J Am Vet Med Assoc. (1995) 206:1721–8. doi: 10.2460/javma.1995.206.11.1721, PMID: 7782244

[9] Pákozdy Á Leschnik M Tichy A Thalhammer J. Retrospective clinical comparison of idiopathic versus symptomatic epilepsy in 240 dogs with seizures. Acta Vet Hung. (2008) 56:471–83. doi: 10.1556/avet.56.2008.4.5, PMID: 19149102

[10] Ghormley TM Feldman DG Cook JR. Epilepsy in dogs five years of age and older: 99 cases (2006–2011). J Am Vet Med Assoc. (2015) 246:447–50. doi: 10.2460/javma.246.4.447, PMID: 25632819

[11] Bush WW Barr CS Darrin EW Shofer FS Vite CH Steinberg SA. Results of cerebrospinal fluid analysis, neurologic examination findings, and age at the onset of seizures as predictors for results of magnetic resonance imaging of the brain in dogs examined because of seizures: 115 cases (1992–2000). J Am Vet Med Assoc. (2002) 220:781–4. doi: 10.2460/javma.2002.220.781, PMID: 11918271

[12] Smith PM Talbot CE Jeffery ND. Findings on low-field cranial MR images in epileptic dogs that lack interictal neurological deficits. Vet J. (2008) 176:320–5. doi: 10.1016/j.tvjl.2007.03.003, PMID: 17499532

[13] Schwartz M Muñana KR Nettifee-Osborne J. Assessment of the prevalence and clinical features of cryptogenic epilepsy in dogs: 45 cases (2003–2011). J Am Vet Med Assoc. (2013) 242:651–7. doi: 10.2460/javma.242.5.651, PMID: 23402412

[14] Arrol L Penderis J Garosi L Cripps P Gutierrez-Quintana R Gonçalves R. Aetiology and long-term outcome of juvenile epilepsy in 136 dogs. Vet Rec. (2012) 170:335. doi: 10.1136/vr.100316, PMID: 22266685

[15] Hall R Labruyere J Volk H Cardy TJ. Estimation of the prevalence of idiopathic epilepsy and structural epilepsy in a general population of 900 dogs undergoing MRI for epileptic seizures. Vet Rec. (2020) 187:e89. doi: 10.1136/vr.105647, PMID: 32303666

[16] Hori M Hagiwara A Goto M Wada A Aoki S. Low-field magnetic resonance imaging: its history and renaissance. Investig Radiol. (2021) 56:669–79. doi: 10.1097/RLI.0000000000000810, PMID: 34292257 PMC8505165

[17] Monteiro R Adams V Keys D Platt SR. Canine idiopathic epilepsy: prevalence, risk factors and outcome associated with cluster seizures and status epilepticus. J Small Anim Pract. (2012) 53:526–30. doi: 10.1111/j.1748-5827.2012.01251.x, PMID: 22835069

[18] Cagnotti G Ferrini S Ala U Bellino C Corona C Dappiano E. Analysis of early assessable risk factors for poor outcome in dogs with cluster seizures and status epilepticus. Front Vet Sci. (2020) 7:7. doi: 10.3389/fvets.2020.575551, PMID: 33195572 PMC7581674

[19] Fentem R de Stefani A Quintana RG Alcoverro E Jones GMC Amengual-Batle P. Risk factors associated with short-term mortality and recurrence of status epilepticus in dogs. J Vet Intern Med. (2022) 36:656–62. doi: 10.1111/jvim.16353, PMID: 34994484 PMC8965210

[20] Zimmermann R Hülsmeyer V-I Sauter-Louis C Fischer A. Status epilepticus and epileptic seizures in dogs. J Vet Intern Med. (2009) 23:970–6. doi: 10.1111/j.1939-1676.2009.0368.x, PMID: 19737178

[21] Shoop-Worrall SJ O'Neill DG Viscasillas J Brodbelt DC. Mortality related to general anaesthesia and sedation in dogs under UK primary veterinary care. Vet Anaesth Analg. (2022) 49:433–42. doi: 10.1016/j.vaa.2022.03.006, PMID: 35985925

[22] Bernasconi A Cendes F Theodore WH Gill RS Koepp MJ Hogan RE. Recommendations for the use of structural magnetic resonance imaging in the care of patients with epilepsy: a consensus report from the international league against epilepsy neuroimaging task force. Epilepsia. (2019) 60:1054–68. doi: 10.1111/epi.15612, PMID: 31135062

[23] Westbrook C Roth CK Talbot J. Instrumentation and Equipment' in MRI in practice. 4th ed. West Sussex, UK: Wiley-Blackwell (2012).

[24] Nagendran A McConnell JF De Risio L José-López R Quintana RG Robinson K. Peri-ictal magnetic resonance imaging characteristics in dogs with suspected idiopathic epilepsy. J Vet Intern Med. (2021) 35:1008–17. doi: 10.1111/jvim.16058, PMID: 33559928 PMC7995424

[25] Suñol A Garcia-Pertierra S Faller KME. Cerebrospinal fluid analysis in dogs: Main patterns and prevalence of albuminocytological dissociation. Vet Rec. (2021) 188:e27. doi: 10.1002/vetr.27, PMID: 33666999

[26] Hasegawa D Saito M Kitagawa M. Neurosurgery in canine epilepsy. Vet J. (2022) 285:105852. doi: 10.1016/j.tvjl.2022.105852, PMID: 35716888

[27] Schwartz M Lamb CR Brodbelt DC Volk HA. Canine intracranial neoplasia: clinical risk factors for development of epileptic seizures. J Small Anim Pract. (2011) 52:632–7. doi: 10.1111/j.1748-5827.2011.01131.x, PMID: 21954970

[28] Rossmeisl J Pancotto T. Intracranial neoplasia and secondary pathological effects In: Platt S Garosi L, editors. Small Animal Neurological Emergencies. Barcelona: Manson Publishing (2012). 461–78.

[29] Miller AD Miller CR Rossmeisl JH. Canine primary intracranial Cancer: a Clinicopathologic and comparative review of glioma, meningioma, and choroid plexus tumors. Front Oncol. (2019) 9:1151. doi: 10.3389/fonc.2019.01151, PMID: 31788444 PMC6856054

[30] Beghi E. The epidemiology of epilepsy. Neuroepidemiology. (2019) 54:185–91. doi: 10.1159/000503831, PMID: 31852003

[31] O’Neill DG Church DB McGreevy PD Thomson PC Brodbelt DC. Longevity and mortality of owned dogs in England. Vet J. (2013) 198:638–43. doi: 10.1016/j.tvjl.2013.09.020, PMID: 24206631

[32] Loncarica T Balducci F Bernardini M. Prevalence of idiopathic epilepsy and structural epilepsy in 74 boxer dogs in a referral hospital. Front Vet Sci. (2022) 9:956648. doi: 10.3389/fvets.2022.956648, PMID: 36061109 PMC9437913

[33] Charalambous M Bhatti SFM Volk HA Platt S. Defining and overcoming the therapeutic obstacles in canine refractory status epilepticus. Vet J. (2022) 283-284:105828. doi: 10.1016/j.tvjl.2022.105828, PMID: 35429630

[34] van Lanen R Colon AJ Wiggins CJ Hoeberigs MC Hoogland G Roebroeck A. Ultra-high field magnetic resonance imaging in human epilepsy: a systematic review. Neuroimage Clin. (2021) 30:102602. doi: 10.1016/j.nicl.2021.102602, PMID: 33652376 PMC7921009

[35] Arnold TC Freeman CW Litt B Stein JM. Low-field MRI: clinical promise and challenges. J Magn Reson Imaging. (2023) 57:25–44. doi: 10.1002/jmri.28408, PMID: 36120962 PMC9771987

[36] Fentem R Nagendran A Marioni-Henry K Madden M Phillipps S Cooper C. Complications associated with cerebrospinal fluid collection in dogs. Vet Rec. (2023) 193:e2787. doi: 10.1002/vetr.2787, PMID: 36906911

[37] Gilbert SE Cardy TJ Bertram S Taylor-Brown F. Diagnostic utility of cerebrospinal fluid analysis in dogs with suspected idiopathic epilepsy. Aust Vet J. (2021) 99:1–5. doi: 10.1111/avj.13018, PMID: 32893907

[38] Coelho AM Maddox TW Sanchez-Masian D Gonçalves R. Diagnostic value of cerebrospinal fluid analysis in a population of dogs with suspected idiopathic epilepsy. Vet Rec. (2019) 185:539. doi: 10.1136/vr.105438, PMID: 31409750

[39] Gonçalves R Anderson TJ Innocent G Penderis J. Effect of seizures on cerebrospinal fluid analysis in dogs with idiopathic epilepsy. Vet Rec. (2010) 166:497–8. doi: 10.1136/vr.b4812, PMID: 20400742

[40] Packer RMA Berendt M Bhatti S Charalambous M Cizinauskas S De Risio L. Inter-observer agreement of canine and feline paroxysmal event semiology and classification by veterinary neurology specialists and non-specialists. BMC Vet Res. (2015) 11:39. doi: 10.1186/s12917-015-0356-2, PMID: 25881213 PMC4337258

[41] Cerda-Gonzalez S Packer RA Garosi L Lowrie M Mandigers PJJ O'Brien DP. International veterinary canine dyskinesia task force ECVN consensus statement: terminology and classification. J Vet Intern Med. (2021) 35:1218–30. doi: 10.1111/jvim.16108, PMID: 33769611 PMC8162615

[42] Mandigers PJJ Santifort KM Lowrie M Garosi L. Canine paroxysmal dyskinesia—a review. Front Vet Sci. (2024) 11:11. doi: 10.3389/fvets.2024.1441332, PMID: 39119350 PMC11308868

[43] Lyon E Pochat H Blot S Troupel T Van Caenegem N Besnard S. Use of video-electroencephalography as a first-line examination in veterinary neurology: development and standardization of electroencephalography in unsedated dogs and cats. Front Vet Sci. (2024) 11:1326165. doi: 10.3389/fvets.2024.1326165, PMID: 38343449 PMC10853351

